# Awareness and use of nicotine pouches in a nationwide sample of adults in Poland

**DOI:** 10.18332/tid/192522

**Published:** 2024-09-09

**Authors:** Mateusz Jankowski, Vaughan W. Rees

**Affiliations:** 1 Department of Population Health, School of Public Health, Centre of Postgraduate Medical Education, Warsaw, Poland; 2 Center for Global Tobacco Control, Department of Social and Behavioral Sciences, Harvard T.H. Chan School of Public Health, Boston, United States

**Keywords:** nicotine pouches, noncombustible products, nicotine addiction, tobacco control policy, emerging nicotine-containing products

## Abstract

**INTRODUCTION:**

Nicotine pouches are a new type of nicotine-containing product that have been marketed in many countries worldwide, generating growing acceptance among consumers. The aim of this study was to assess factors associated with public awareness and use of nicotine pouches among adults in Poland.

**METHODS:**

A cross-sectional survey was conducted with a nationally representative sample of 1080 adults in Poland (February 2024). Awareness of nicotine pouches, history of use, current (past 30-day) use of nicotine pouches, as well as perception of harm was assessed using a purpose-designed questionnaire.

**RESULTS:**

Awareness of nicotine pouches was reported by 24% of the sample, while 9.2% reported ever having used a nicotine pouch product, and 4.3% using a nicotine pouch in the past 30 days. Among all respondents, 60.7% perceived nicotine pouches as harmful as combustible cigarettes, 28.2% perceived nicotine pouches as less harmful, and 11% as more harmful than combustible cigarettes. In multivariable logistic regression model, women (adjusted odds ratio, AOR=1.40; 95% CI: 1.03–1.91; p<0.05), individuals aged <60 years (p<0.05), current tobacco smokers (AOR=2.59; 95% CI: 1.75–3.82; p<0.001), former tobacco smokers (AOR=1.53; 95% CI: 1.01–2.32; p<0.05) and ever users of e-cigarette or heated tobacco (AOR=2.90; 95% CI: 2.07–4.05; p<0.001) were more likely to declare that had ever heard of nicotine pouches. Moreover, individuals aged <60 years (p<0.05), occupationally active individuals (AOR=1.82; 95% CI: 1.01–3.31; p<0.05), current tobacco smokers (AOR=2.71; 95% CI: 1.48–4.97; p<0.01), and ever users of e-cigarette or heated tobacco (AOR=5.29; 95% CI: 2.96–9.44; p<0.001) were more likely to declare ever use of nicotine pouches.

**CONCLUSIONS:**

This study provides the first national data on public awareness and use (ever and current) of nicotine pouches in Poland. Young adults, current smokers, and ever e-cigarette and heated tobacco users are at higher risk of ever use of nicotine pouches, so policy interventions are needed to protect young people from nicotine pouch marketing and nicotine initiation.

## INTRODUCTION

Smokeless tobacco (SLT) has a long history of use and is still used by 300 million people globally^1^. In many developed countries, SLT is marketed in a wide range of forms, including chewing tobacco, moist snuff, snus and dissolvable tobacco products^1,2^. Although the health risks of SLT are generally considered to be lower than for combusted tobacco products, their use is associated with increased risk of certain cancers, particularly those of the head and neck, as well as cardiovascular disease and dental diseases^3^. Newer SLT products, including Swedish-style snus and dissolvable tobacco have been marketed as lower risk alternatives that are easy to conceal and free of emissions, which has raised interest among youth^4^. Over the past two decades, snus in pouched form attained new interest among younger consumers as new products were introduced and marketed to youth, some of which were produced by cigarette manufacturers with popular cigarette brand names (e.g. Camel Snus).

Recently, a new variant of pouched product has been marketed under brand names such as Zyn, Velo, On!, ROGUE, and NIIN^5-7^. So-called nicotine pouches do not contain tobacco but provide nicotine in a substrate of white inert cellulose powder, flavorings, humectants, acidity regulators, and stabilizers^5,8,9^. As with other pouched tobacco products, nicotine pouches are placed between gum and lip where nicotine is absorbed through the oral mucosa^6,10^. Unlike some snus products, nicotine pouches do not require spitting during use^5,10^. The total nicotine dose in nicotine pouches ranges usually from 2 to 20 mg per pouch, and is comparable to that of popular snus products^11^. They are sold in a range of flavors, including tobacco, menthol, mint, fruity, coffee, and spices^5,12^.

Toxicological analyses have shown that nicotine pouches contain fewer toxic compounds, and lower concentrations of toxic compounds than comparable products such as snus^8^. While the health risks of nicotine pouches have not been fully evaluated, the risks may be more relevant to the potential for nicotine dependence rather than chronic diseases associated with tobacco exposure^13^. These products may have the potential to reduce health risks of adult smokers if smokers switch completely^1,2,8^. However, the pharmacokinetics of nicotine delivered by oral tobacco products and the lack of smoking-related behaviors can make complete switching a challenge for smokers who have become accustomed to a highly specific form of nicotine dosing to support a nicotine use disorder^14^. Dual use of nicotine pouches and other nicotine-containing products (combusted cigarettes, electronic cigarettes) may increase exposure to nicotine and lead to more severe nicotine dependence, without lowering health risks^15^. Of particular concern is the marketing of nicotine pouch products to youth who would not otherwise use tobacco products^4,5,8^.

Poland is a country with a high prevalence of nicotine product use^16^. In February 2024, among adults in Poland, 24.5% were daily smokers, 5.9% were daily e-cigarette users and 4.9% were daily heated tobacco users^16^. There is also a high prevalence of past 30-day e-cigarette use among adolescents in Poland reaching one-third of adolescents aged 15–19 years^17^. In Poland, nicotine pouches are regulated as smokeless tobacco products^10^. Nicotine pouches are widely promoted at points-of-sale including product location at eye level, and advertising banners that highlight flavor variants and price^18^. Nicotine pouches are also promoted on the Internet, including discount coupons lowering the price of products and the possibility of ordering a test package containing various flavors of pouches^7,18^.

While the prevalence of snus and other SLT use in Poland is <1% of the population aged ≥15 years^19^, there is a lack of data on the prevalence of nicotine pouch use. Accurate and timely data are needed to assess knowledge and attitudes toward oral nicotine products, especially among adult smokers who might consider switching to a lower risk alternative, and youth, whose use may lead to problems of nicotine dependence^20^. Regulatory strategies aimed at restricting appeal among youth, including health communication and restrictions on sale and marketing, require an evidence base to inform policy approaches. The aim of this study was to assess factors associated with public awareness and use of nicotine pouches among adults in Poland.

## METHODS

### Study design and sampling

A cross-sectional survey was conducted with a nationally representative sample of 1080 adults in Poland. Data were collected in February 2024 by a specialized survey company^21^ using a computer-assisted web interview technique, as part of the ‘Poles’ attitudes towards smoking’ survey^16^. Participants were recruited from an online panel with >100000 verified individuals^21^, to form a representative sample of the Polish adult population with respect to demographic characteristics. Sample selection was carried out in two stages. In the first step, the population was segregated into demographic (gender, age, regions) subgroups based on mutual exclusivity. In the second step, respondents were selected based on quota sampling and stratification model. The stratification model was based on demographic data from the National Population Report^22^ and includes gender, age as well as household size, and territorial distribution within administrative units. Participants were considered eligible if aged ≥18 years, and ineligible if they had no access to the internet. Every selected participant received a personalized e-mail with a URL to the study questionnaire and a text message with a reminder. The URL granted each invitee single access to the survey. If selected respondents refused to take part in the study, a new respondent was invited based on the demographic criteria. The study questionnaire was administered in an online format that required participants to complete all the fields before the closure of the questionnaire. The overall response rate was approximately 25%. Participation in the study was voluntary and anonymous. Informed consent was obtained before the survey procedure commenced.

All procedures followed the Declaration of Helsinki and the study protocol was approved by the Ethics Review Board at the Centre of Postgraduate Medical Education, Warsaw, Poland (document number 403/2023).

### Measures

Discussions with public health scientists, a field survey of the retail environment and websites were carried out to ascertain nicotine pouch brands currently marketed for sale in Poland, which should be included in the study questionnaire. Velo, Lyft, Zyn, On! were identified as widely marketed by tobacco companies, and these brands were used in the questionnaire. In Poland, there is a limited availability of snus, and there is no broad marketing of snus brands in Poland. A pilot study with 12 adults was conducted to verify the questions used in the questionnaire. In the study questionnaire, pictures were not provided because we were encouraged for ethical reasons to avoid unintended promotion of nicotine products.

Awareness was measured using the following question: ‘Have you ever heard of nicotine pouches (e.g. Velo, Lyft, Zyn, On!)?’ (yes/no). History of use was measured using the following question: ‘Have you ever used nicotine pouches (e.g. Velo, Lyft, Zyn, On!), even once?’ (yes/no). Current (past 30-day) use of nicotine pouches was measured using the following question: ‘Do you currently (in the last 30 days) use nicotine pouches (e.g. Velo, Lyft, Zyn, On!)?’ with three possible answers: yes, daily; yes, occasionally; no. Those who indicated daily or occasional use of nicotine pouches in the last 30 days were assigned to the group of past 30-day nicotine pouch users.

Perception of harm was measured using the following question: ‘Compared to combustible cigarettes, how harmful do you think are: 1) nicotine pouches; and 2) snus?’ with three possible answers: less harmful, same harmful, more harmful. Respondents were also asked about ever and current (past 30-day) smoking, e-cigarette use, and heated tobacco use^16^.

*Sociodemographics*

Sociodemographic data collected included: gender, age, education level (having a university degree), occupational status, household (all the related people living together in a house or flat) income class, number of household members, and region of residence. Active occupational status included employees and self-employed; passive occupational status included the unemployed, students, or pensioners/retirees (people who have temporarily or permanently left the workforce owing to age or disability). Household income was assessed with the question: ‘How do you assess your own/your family's financial situation (high/medium/low)?’. Region of residence was defined by the official administrative definition of rural and urban (residence in a city regardless of the number of inhabitants).

### Statistical analysis

The distribution of categorical variables was shown by frequencies and proportions. Categorical variables were compared using the cross-tabulation and independent samples two-tailed chi-squared test. The univariable and multivariable logistic regression analyses were conducted to calculate the odds ratios (ORs) and 95% confidence intervals (CIs) of selected variables in relation to: 1) ever heard of nicotine pouches; and 2) ever use of nicotine pouches. The statistical significance level was based on the criterion p<0.05.

Associations between personal characteristics (gender, age), socioeconomic status (occupational status and household income class), smoking status (tobacco smoking in the last 30 days), and novel noncombusted products use (ever e-cigarette or heated tobacco use) with 1) ever heard of nicotine pouches, and 2) ever use of nicotine pouches, were conducted using logistic regression analyses. Model 1 includes demographic covariates (including gender and age) to assess their association with: 1) ever heard of nicotine pouches; and 2) ever use of nicotine pouches, conducted using logistic regression analyses. Model 2 adds occupational status and household income class (a proxy for socio-economic position). Model 3 adds smoking status (tobacco smoking in the last 30 days). Model 4 adds ever non-combusted products use (e-cigarettes or heated tobacco products). Data were analyzed with SPSS, version 28 (IBM, Armonk, NY, USA).

## RESULTS

Analyses were carried out on responses from 1080 adult individuals aged ≥18 years (53% women). Table 1 shows the characteristics of the study sample. Awareness of nicotine pouches was reported by 24% of the sample, while 9.2% reported ever having used a nicotine pouch product, and 4.3% using a nicotine pouch in the past 30 days (Table 2). Among all respondents, 60.7% perceived nicotine pouches as harmful as combustible cigarettes, 28.2% perceived nicotine pouches as less harmful and 11% as more harmful than combustible cigarettes (Table 1). Among smokers, 33.5% perceived nicotine pouches as less harmful than cigarettes, and 57.0% rated nicotine pouches to be as harmful as combustible cigarettes. Among former smokers, 28.3% perceived nicotine pouches as less harmful than combustible cigarettes, 10.2% perceived them as more harmful, and 61.5% rated nicotine pouches to be as harmful as combustible cigarettes. Among never smokers, 24.3% perceived nicotine pouches as less harmful than combustible cigarettes, 12.7% perceived them as more harmful, and 62.9% rated nicotine pouches to be as harmful as combustible cigarettes (p=0.06).

**Table 1:** Characteristics of the study population (N=1080)

*Characteristics*	*n (%)*
**Gender**	
Men	508 (47.0)
Women	572 (53.0)
**Age** (years)	
18–29	140 (13.0)
30–59	615 (56.9)
≥60	325 (30.1)
**Education level**	
Tertiary – university degree	469 (43.4)
Lower than tertiary – primary, secondary, or vocational school	611 (56.6)
**Occupational status**	
Active (currently employed)	656 (60.7)
Passive (not currently employed)	424 (39.3)
**Household income class**	
High	330 (30.6)
Medium	606 (56.1)
Low	144 (13.3)
**Number of household members**	
1	134 (12.4)
≥2	946 (87.6)
**Region of residence**	
Rural	416 (38.5)
Urban	664 (61.5)
**Nicotine product use behaviors**	
Current tobacco smoking	328 (30.4)
Former smokers	304 (28.1)
Never smokers	448 (41.5)
Ever e-cigarette use	356 (33.0)
Current e-cigarette use	164 (15.2)
Ever heated tobacco use	223 (20.6)
Current heated tobacco use	118 (10.9)
**Harm perception of nicotine pouches compared to combustible cigarettes**	
Less harmful	305 (28.2)
As harmful	656 (60.7)
More harmful	119 (11.0)

**Table 2:** Public awareness and use of nicotine pouches among adults in Poland, 2024 (N=1080)

*Variables*	*Total n*	*Ever heard of nicotine pouches*			*Ever use of nicotine pouches*			*Current (past 30-day) use of nicotine pouches*		
		*n*	*% (95% CI)*	p*	*n*	*% (95% CI)*	p*	*n*	*% (95% CI)*	p*
**Overall**	1080	259	24.0 (21.5–26.6)		99	9.2 (7.6–11.0)		46	4.3 (3.2–5.6)	
**Gender**										
Men	508	107	21.1 (17.7–24.8)	**0.03**	50	9.8 (7.6–12.7)	0.5	28	5.5 (3.8–7.9)	0.06
Women	572	152	26.6 (23.1–30.3)		49	8.6 (6.5–11.2)		18	3.1 (2.0–4.9)	
**Age** (years)										
18–29	140	54	38.6 (30.9–46.8)	**<0.001**	24	17.1 (11.8–24.2)	**<0.001**	8	5.7 (2.9–10.9)	**<0.001**
30–59	615	160	26.0 (22.7–29.6)		68	11.1 (8.8–13.8)		36	5.9 (4.3–8.0)	
≥60	325	45	13.8 (10.5–18.0)		7	2.2 (1.1–4.4)		2	0.6 (0.2–2.2)	
**Education level**										
Tertiary	469	108	23.0 (19.5–27.1)	0.5	43	9.2 (6.9–12.1)	0.9	22	4.7 (3.1–7.0)	0.5
Lower than tertiary	611	151	24.7 (21.5–28.3)		56	9.2 (7.1–11.7)		24	3.9 (2.7–5.8)	
**Occupational status**										
Active	656	176	26.8 (23.6–30.4)	**0.01**	81	12.3 (10.1–15.1)	**<0.001**	41	6.3 (4.6–8.4)	**<0.001**
Passive	424	83	19.6 (16.1–23.6)		18	4.2 (2.7–6.6)		5	1.2 (0.5–2.7)	
**Household income class**										
High	330	82	24.8 (20.5–29.8)	0.05	32	9.7 (7.0–13.4)	0.6	15	4.5 (2.8–7.4)	0.6
Medium	606	132	21.8 (18.7–25.2)		51	8.4 (6.5–10.9)		23	3.8 (2.5–5.6)	
Low	144	45	31.3 (24.3–39.2)		16	11.1 (7.0–17.3)		8	5.6 (2.8–10.6)	
**Number of household members**										
1	134	16	11.9 (7.5–18.5)	**<0.001**	12	9.0 (5.2–15.0)	0.9	3	2.2 (0.8–6.4)	0.2
≥2	946	243	25.7 (23.0–28.6)		87	9.2 (7.5–11.2)		43	4.5 (3.4–6.1)	
**Region of residence**										
Rural	416	101	24.3 (20.4–28.6)	0.9	33	7.9 (5.7–10.9)	0.3	13	3.1 (1.8–5.3)	0.1
Urban	664	158	23.8 (20.7–27.2)		66	9.9 (7.9–12.5)		33	5.0 (3.6–6.9)	
**Former smokers**										
Yes	304	62	20.4 (16.3–25.3)	0.8	22	7.2 (4.8–10.7)	0.2	3	1.0 (0.3–2.9)	**<0.001**
No	776	197	25.4 (22.5–28.6)		77	9.9 (8.0–12.2)		43	5.5 (4.1–7.4)	
**Tobacco smoking status**										
Current	328	128	39.0 (33.9–44.4)	**<0.001**	58	17.7 (13.9–22.2)	**<0.001**	40	12.2 (9.1–16.2)	**<0.001**
Former	304	62	20.4 (16.3–25.3)		22	7.2 (4.8–10.7)		3	1.0 (0.3–2.9)	
Never	448	69	15.4 (12.4–19.0)		19	4.2 (2.7–6.5)		3	0.7 (0.2–2.0)	
**Ever e-cigarette use**										
Yes	356	157	44.1 (39.0–49.3)	**<0.001**	76	21.3 (17.4–25.9)	**<0.001**	39	11.0 (8.1–14.6)	**<0.001**
No	724	102	14.1 (11.7–16.8)		23	3.2 (2.1–4.7)		7	1.0 (0.5–2.0)	
**Current e-cigarette use**										
Yes	164	86	52.4 (44.8–59.9)	**<0.001**	54	32.9 (26.2–40.4)	**<0.001**	35	21.3 (15.8–28.2)	**<0.001**
No	916	173	18.9 (16.5–21.6)		45	4.9 (3.7–6.5)		11	1.2 (0.7–2.1)	
**Ever heated tobacco use**										
Yes	223	116	52.0 (45.5–58.5)	**<0.001**	61	27.4 (21.9–33.6)	**<0.001**	38	17.0 (12.7–22.5)	**<0.001**
No	857	143	16.7 (14.3–19.3)		38	4.4 (3.3–6.0)		8	0.9 (0.5–1.8)	
**Current heated tobacco use**										
Yes	118	78	66.1 (57.2–74.0)	**<0.001**	47	39.8 (31.5–48.9)	**<0.001**	37	31.4 (23.7–40.2)	**<0.001**
No	962	181	18.8 (16.5–21.4)		52	5.4 (4.2–7.0)		9	0.9 (0.5–1.8)	
**Harm perception of snus compared to combustible cigarettes**										
Less harmful	354	115	32.5 (27.8–37.5)	**<0.001**	54	15.3 (11.9–19.4)	**<0.001**	30	8.5 (6.0–11.8)	**<0.001**
As harmful	616	124	20.1 (17.2–23.5)		37	6.0 (4.4–8.2)		13	2.1 (1.2–3.6)	
More harmful	110	20	18.2 (12.1–26.4)		8	7.3 (3.7–13.7)		3	2.7 (0.9–7.7)	
**Harm perception of nicotine pouches compared to combustible cigarettes**										
Less harmful	305	117	38.4 (33.1–43.9)	**<0.001**	50	16.4 (12.7–21.0)	**<0.001**	32	10.5 (7.5–14.4)	**<0.001**
As harmful	656	115	17.5 (14.8–20.6)		40	6.1 (4.5–8.2)		11	1.7 (0.9–3.0)	
More harmful	119	27	22.7 (16.1–31.0)		9	7.6 (4.0–13.8)		3	2.5 (0.9–7.2)	

* Independent samples two-tailed chi-squared test.

Women were more likely to report awareness of nicotine pouch products than men (26.6% vs 21.1%; p=0.03), and younger respondents (aged 18–29 years) were more likely to report awareness than older respondents (Table 2). Individuals aged 30–59 years were most likely to report ever having used a nicotine pouch (5.9%; p<0.001). Likewise, those who are currently employed, people who currently smoke, and people who have ever or currently use an e-cigarette or heated tobacco product were more likely to report awareness, past use or current use of nicotine pouches. Similarly, those who perceived snus or nicotine pouches as less harmful compared to combustible cigarettes, were also more likely to report awareness, past use or current use of nicotine pouches (Table 2).

The results of the univariable and multivariable logistic regression analyses are presented in Tables 3 and 4. When adjusted for demographic covariates, socio-economic position, smoking status and ever non-combusted products use, women (AOR=1.40; 95% CI: 1.03–1.91; p<0.05), individuals aged <60 years (p<0.05), current tobacco smokers (AOR=2.59; 95% CI: 1.75–3.82; p<0.001), former tobacco smokers (AOR=1.53; 95% CI: 1.01–2.32; p<0.05) and ever users of e-cigarette or heated tobacco (AOR=2.90; 95% CI: 2.07–4.05; p<0.001) were more likely to declare that had ever heard of nicotine pouches (Table 3). Moreover, individuals aged <60 years (p<0.05), occupationally active individuals (AOR=1.82; 95% CI: 1.01–3.31; p<0.05), current tobacco smokers (AOR=2.71; 95% CI: 1.48–4.97; p<0.01), and ever users of e-cigarette or heated tobacco (AOR=5.29; 95% CI: 2.96–9.44; p<0.001) were more likely to declare ever use of nicotine pouches (Table 4).

**Table 3:** Factors associated with public awareness of nicotine pouches among adults in Poland, 2024 (N=1080)

*Variables*	*Univariable logistic regression*	*Multivariable logistic regression*			
		*Model 1*	*Model 2*	*Model 3*	*Model 4*
	*OR (95%CI)*	*AOR (95%CI)*			
**Gender**					
Male ®	1	1	1	1	1
Female	1.36 (1.02–1.80)*	1.29 (0.96–1.71)	1.30 (0.97–1.73)	1.46 (1.08–1.98)*	1.40 (1.03–1.91)*
**Age** (years)					
18–29	3.91 (2.46–6.21)***	3.80 (2.39–6.05)***	3.50 (2.15–5.72)***	4.46 (2.66–7.47)***	2.76 (1.61–4.76)***
30–59	2.19 (1.52–3.15)***	2.17 (1.51–3.12)***	1.96 (1.31–2.95)**	2.06 (1.35–3.15)***	1.79 (1.16–2.77)**
≥60 ®	1	1	1	1	1
**Occupational status**					
Active	1.51 (1.12–2.03)**		1.17 (0.83–1.64)	1.16 (0.82–1.66)	1.05 (0.73–1.51)
Passive ®	1		1	1	1
**Household income class**					
High ®	1		1	1	1
Medium	0.84 (0.61–1.16)		0.95 (0.69–1.32)	0.94 (0.67–1.31)	0.95 (0.67–1.34)
Low	1.38 (0.89–2.17)		1.47 (0.94–2.29)	1.36 (0.86–2.16)	1.37 (0.85–2.19)
**Smoking status**					
Current	3.52 (2.50–4.94)***			4.20 (2.94–5.99)***	2.59 (1.75–3.82)***
Former	1.41 (0.96–2.06)			1.99 (1.33–2.98)***	1.53 (1.01–2.32)*
Never ®	1			1	1
**Ever use of e-cigarette or heated tobacco**					
Yes	4.58 (3.41–6.16)***				2.90 (2.07–4.05)***
No ®	1				1

Model 1: includes demographic covariates (including gender and age) to assess associations with awareness of nicotine pouches. Model 2: adds occupational status and household income class (a proxy for socio-economic position). Model 3: adds smoking status (tobacco smoking in the last 30 days). Model 4: adds ever non-combusted products use (e-cigarettes or heated tobacco products).

* p<0.05.

** p<0.01.

*** p<0.001.

® Reference categories.

**Table 4:** Factors associated with ever use of nicotine pouches among adults in Poland, 2024 (N=1080)

*Variables*	*Univariable logistic regression*	*Multivariable logistic regression*			
		*Model 1*	*Model 2*	*Model 3*	*Model 4*
	*OR (95%CI)*	*AOR (95%CI)*			
**Gender**					
Male ®	1	1	1	1	1
Female	0.86 (0.57–1.30)	0.78 (0.51–1.19)	0.82 (0.54–1.25)	0.92 (0.59–1.42)	0.82 (0.53–1.29)
**Age** (years)					
18–29	9.40 (3.94–22.40)***	9.72 (4.07–23.22)***	7.18 (2.90–17.72)***	9.31 (3.71–23.39)***	4.91 (1.91–12.60)***
30–59	5.65 (2.56–12.45)***	5.72 (2.59–12.60)***	3.91 (1.69–9.05)**	4.04 (1.73–9.47)**	3.33 (1.41–7.91)**
≥60 ®	1	1	1	1	1
**Occupational status**					
Active	3.18 (1.88–5.38)***		2.03 (1.15–3.60)*	2.11 (1.18–3.79)*	1.82 (1.01–3.31)*
Passive ®	1		1	1	1
**Household income class**					
High ®	1		1	1	1
Medium	0.86 (0.54–1.36)		1.03 (0.64–1.66)	1.00 (0.61–1.64)	1.03 (0.62–1.71)
Low	1.16 (0.62–2.20)		1.36 (0.70–2.62)	1.21 (0.61–2.38)	1.22 (0.61–2.44)
**Smoking status**					
Current	4.85 (2.83–8.32)***			5.71 (3.26–9.98)***	2.71 (1.48–4.97)**
Former	1.76 (0.94–3.31)			2.71 (1.41–5.21)**	1.74 (0.88–3.43)
Never ®	1			1	1
**Ever use of e-cigarette or heated tobacco**					
Yes	9.34 (5.51–15.83)***				5.29 (2.96–9.44)***
No ®	1				1

Model 1: includes demographic covariates (including gender and age) to assess associations with ever use of nicotine pouches. Model 2: adds occupational status and household income class (a proxy for socio-economic position). Model 3: adds smoking status (tobacco smoking in the last 30 days). Model 4: adds ever non-combusted products use (e-cigarettes or heated tobacco products).

* p<0.05.

** p<0.01.

*** p<0.001.

® Reference categories.

## DISCUSSION

This study revealed that 24% of adults in Poland had heard of nicotine pouches before, and 9.2% had ever tried nicotine pouches. The percentage of current users of nicotine pouches was 4.3% of the adult population. Nicotine pouches were perceived as less harmful compared to combustible cigarettes by 28.2% of respondents. There were sociodemographic differences in the percentage of individuals who ever heard or used (ever or current) nicotine pouches. Women were more likely to ever hear of nicotine pouches. Younger adults, current smokers, and ever e-cigarette or heated tobacco users were more likely to ever hear of as well as ever used nicotine pouches. This is the first study on nicotine pouches awareness and use in Poland.

Nicotine pouches are present on the market since 2016 and widely promoted in Europe since 2022^8,10,23^. In 2021, the prevalence of nicotine pouches in the European Union (EU) was estimated at 0.3% of the adult population^23^. There are limited national data on the awareness of nicotine pouches and the prevalence of use in Europe. Havermans et al.^24^ reported that in 2020, 6.88% of adolescents and adults in The Netherlands ever heard of nicotine pouches, 0.56% ever used and 0.06% were current users. However, 9.09% of adolescents aged 13–17 years declared awareness of nicotine pouches. The highest percentage of individuals who ever used nicotine pouches (1.34%) was among adults aged 25–44 years^24^. Tattan-Birch et al.^25^ reported that between 2020 and 2021, the prevalence of use of nicotine pouches among adults in Great Britain increased from 0.14% to 0.32%. In this study carried out in 2024, 24% of adults in Poland ever heard of nicotine pouches which is almost four times higher than reported by Havermans et al.^24^. However, in our study data from the adolescents were not collected. The prevalence of ever and current use of nicotine pouches observed in this study (9.2% and 4.3% of the adult population, respectively) was several times higher than previously reported in The Netherlands or Great Britain^24,25^. The prevalence of current use of nicotine pouches in Poland (4.3%) was comparable to that of current e-cigarette (5.9%) and heated tobacco (4.9%) users.

Morean et al.^26^ assessed awareness, and use of oral nicotine pouches among young adults (aged 18–25 years) in the United States (US)^26^. In 2021, 41.5% of young adults in the US had heard of nicotine pouches and 10.3% had ever used nicotine pouches^26^. Similar results were reported by Kramer et al.^27^. Based on the 2021 National Tobacco Youth Survey among middle and high school students, Kramer et al.^27^ reported that 35.5% of students had ever heard of nicotine pouches, 1.9% reported ever using and 0.8% reported current use^27^. In this study, the prevalence of ever heard and ever use of nicotine pouches was also the highest among young adults – 38.6% of adults aged 18–29 years had ever heard of nicotine pouches and 17.1% had ever used them. Findings from this study are in line with data from the US and confirm high awareness and use of nicotine pouches among young people^26,27^.

Flavoring nicotine products, without unpleasant smells are often targeted to women^28^. Hendlin et al.^29^ showed that nicotine pouches manufacturers may also target women. In this study, women were more likely to declare that ever heard of nicotine pouches, which supports the claim of targeting women by the tobacco industry. The opposite results were observed by Havermans et al.^24^ among adolescents and adults in The Netherlands, where the higher awareness of nicotine pouches was among men (8.32%) than women (5.47%). However, in this study, there were no significant gender differences in the prevalence of ever use of nicotine pouches. This is in line with the national data on the lack of gender differences in the prevalence of smoking in Poland, observed in previous years^19^.

Nicotine pouches are marketed as a less risky alternative to combustible tobacco^7,30^. Previously published data from the US, UK, and the Netherlands showed that current smokers or e-cigarette users more often declared awareness and ever use of nicotine pouches compared to those who do not use nicotine products^24,26,27^. Findings from this study also confirmed that current smokers as well as ever e-cigarette or heated tobacco users were more likely to ever hear of or ever use nicotine pouches. Former smokers were also more likely to declare that had ever heard of nicotine pouches, which suggest this group is also vulnerable to marketing of nicotine pouches. In this study, ever use of e-cigarettes or heated tobacco was the most important factor associated with ever heard of nicotine pouches or ever use of nicotine pouches. This finding suggests that individuals who ever tried novel non-combusted products are at higher risk of hearing of nicotine pouches or trying them.

Lessons learned from the vaping epidemic in countries like the US showed that novel non-combusted products, primarily targeted to smokers who want to quit or reduce risk, are often used by young adults, even as a source of nicotine initiation^31^. Findings from this study confirmed that younger adults aged 18–29 years are more likely to hear of nicotine pouches and use them. Nicotine pouches are marketed in social media and offered in different flavors that may attract adolescents, so policy interventions are needed to protect adolescents from exposure to nicotine pouches marketing^5,7,12,30^. Content analysis of nicotine pouches marketing strategies published by Ling et al.^7^ showed that marketing slogans of top nicotine pouches brands include numerous lifestyle claims. Ever use of e-cigarettes or heated tobacco was the most important factor associated with ever heard and use of nicotine pouches, which suggests that individuals who used novel non-combusted products are likely to experiment with other forms of nicotine products, including those marketed as lifestyle products^7^.

Smokeless products are generally less risky than combustible tobacco products when used by adult smokers who want to quit smoking^1,2,23^. In this study, 60% of respondents perceived nicotine pouches as harmful as combustible tobacco, and only 28.2% perceived nicotine pouches as less harmful. This observation may result from a limited general awareness of nicotine pouches as only one-quarter of adults heard of nicotine pouches before. Findings from studies on perceptions of health risks of e-cigarettes have shown that a significant percentage of the population believe that nicotine is a primary cause of harm arising from smoking cigarettes^32,33^. Communication on the health risks of nicotine pouches should be carefully designed to reduce the risk of use of nicotine pouches by non-smokers, especially adolescents. Nicotine pouches are not regulated as a separate category in the EU Tobacco Products Directive^10,23^. Numerous countries, including Poland, are working on national regulations on nicotine pouches^10,23^. Policy interventions in Poland are focused on the classification of nicotine pouches as a separate category of nicotine-containing products, product standardization (including the maximum nicotine dose in the product), remote sales, and taxation. Policy interventions should be focused on the protection of adolescents against marketing and promotion of nicotine pouches as well as product packing and flavoring that may attract minors.

As has been the case for pouched tobacco products, nicotine pouches have been marketed by tobacco manufacturers to adult smokers^7^ and are usually sold in the same retail outlets as tobacco products^5^. A variety of flavors including fruity like cherry, mango, or citrus makes nicotine pouches attractive to youth^4,5,12^. In many countries, nicotine pouches, as novel nicotine-containing products are not regulated or not specifically regulated^10^. Lack of regulation may lead to uncontrolled marketing of nicotine pouches, including marketing to youth^3,10^. There is a lack of EU regulations on the nicotine concentration in the pouches and product design^10,23^. Nicotine concentration and pharmacokinetics impact the change of complete switching or quitting^14,15,32^. Low nicotine products may be insufficient to help adult smokers switch and may support initiation in young people and increase the severity of dependence among adult smokers without supporting full switching^34^. Product regulatory standards are needed.

### Limitations

First, a non-causal design and limitations on the number of questions leading to non-causal design must be considered as a study limitation. This study was limited to the adult population as representative data for the demographic structure of adolescents were not available with CAWI sampling strategy used in this study. Questions were limited to awareness, ever use and current use of nicotine pouches, and we did not collect data on source of knowledge of nicotine pouches or patterns of use (including product type, most common flavors, and the number of pouches consumed daily). There are different methods of assessing public awareness of novel nicotine-containing products, including listing of the most common products or pictures of these products. In the study questionnaire, pictures were not provided because we were encouraged for ethical reasons to avoid unintended promotion of nicotine products. All data were self-reported, raising the potential for recall and demand biases. Simple and multivariable logistic regression models were presented, and weights were not used in regression models. This is the first study on nicotine pouches awareness and use of pouches in Poland and the prevalence of use is relatively small, so we limited our analysis to a basic descriptive approach, without sex specific analyses. There may also be limited generalizability of these data to other countries, owing to different social norms and tobacco control policies in other international settings.

## CONCLUSIONS

This study provides the first national data on public awareness and use (ever and current) of nicotine pouches in Poland. One-quarter of adults in Poland had ever heard of nicotine pouches and almost one-tenth ever tried nicotine pouches which indicates that nicotine pouches are an emerging nicotine product category that requires regulation. Young adults, current smokers, and ever e-cigarette and heated tobacco users are at higher risk of ever use of nicotine pouches, so policy interventions are needed to protect young people from nicotine pouch marketing and nicotine initiation.

## Data Availability

The data supporting this research are available from the authors on reasonable request.
